# Targeting SARS‐CoV‐2 with Chaga mushroom: An in silico study toward developing a natural antiviral compound

**DOI:** 10.1002/fsn3.2576

**Published:** 2021-10-20

**Authors:** Jehane Ibrahim Eid, Biswadeep Das, Majdah Mohamed Al‐Tuwaijri, Wesam Taha Basal

**Affiliations:** ^1^ Department of Zoology Faculty of Science Cairo University Cairo Egypt; ^2^ School of Biotechnology KIIT University Bhubaneswar India; ^3^ Department of Biology Faculty of Applied Science Umm Al‐Qura University Makkah Al‐Mukarramah Saudi Arabia

**Keywords:** beta‐glucan, betulinic acid, docking, galactomannan, *Inonotus obliquus*, SARS‐CoV‐2

## Abstract

The novel coronavirus (SARS‐CoV‐2) has caused large‐scale global outbreaks and mainly mediates host cell entry through the interaction of its spike (S) protein with the human angiotensin‐converting enzyme‐2 (ACE‐2) receptor. As there is no effective treatment for SARS‐CoV‐2 to date, it is imperative to explore the efficacy of new compounds that possess potential antiviral activity. In this study, we assessed the potential binding interaction of the beneficial components of Chaga mushroom, a natural anti‐inflammatory and immune booster with that of the SARS‐CoV‐2 receptor‐binding domain (RBD) using molecular docking, MD simulation, and phylogenetic analysis. Beta glycan, betulinic acid, and galactomannan constituents of Chaga mushroom exhibited strong binding interaction (−7.4 to −8.6 kcal/mol) forming multivalent hydrogen and non‐polar bonds with the viral S1‐carboxy‐terminal domain of the RBD. Specifically, the best interacting sites for beta glycan comprised ASN‐440, SER 373, TRP‐436, ASN‐343, and ARG 509 with average binding energy of −8.4 kcal/mol. The best interacting sites of galactomannan included ASN‐437, SER 373, TRP‐436, ASN‐343, and ALA 344 with a mean binding energy of −7.4 kcal/mol; and the best interacting sites of betulinic acid were ASN‐437, SER 373, TRP‐436, PHE 342, ARG 509, and ALA 344 that strongly interacted with the S‐protein (ΔG = −8.1 kcal/mol). The docking results were also compared with an S‐protein binding analog, NAG and depicted similar binding affinities compared with that of the ligands (−8.67 kcal/mol). In addition, phylogenetic analysis using global isolates depicted that the current SARS‐CoV‐2 isolates possessed a furin cleavage site (NSPRRA) in the RBD, which was absent in the previous isolates that indicated increased efficacy of the present virus for enhanced infection through increased interaction with ACE‐2. The results showed that Chaga could be an effective natural antiviral that can supplement the current anti‐SARS‐CoV‐2 drugs.

## Graphical abstract

Docking analysis revealed that Chaga mushroom components (beta glycan, galactomannan, and betulinic acid) bound to the S1‐CTD residues of SARS‐CoV‐2 involved in ACE‐2‐RBD interaction.

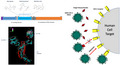

## 1 INTRODUCTION

The recent emergence of a novel severe acute respiratory syndrome corona virus (SARS‐CoV‐2) from China with more than 30 million confirmed cases and 900,000 deaths worldwide has brought a paradigm shift in the global epidemiology (Chen et al., 2020; WHO, 2020). SARS‐CoV‐2 exhibits a range symptoms including mild fever, sore throat, loss of taste and smell, respiratory distress, and fatal multi‐organ failure (Machhi et al., 2020; Sharma et al., 2020; Zimmermann and Curtis, 2020). SARS‐CoV‐2 possesses a unique structural protein, the spike glycoprotein, or S‐protein, which is responsible for aiding entry into the host cell via interaction of the receptor‐binding domain (RBD) with the peptidase domain of the angiotensin‐converting enzyme‐2 (ACE‐2) expressed on the host immune cells (Shang, Wan, Liu, et al., 2020; Wang et al., 2020; Yan et al., 2020). More detailed structural analysis has shown that the RBD comprises certain conservative amino acids, spanning from 326 to 580 amino acids that primarily aid in the S‐protein recognition and binding to ACE‐2 (Tai et al., 2020; Walls et al., 2020; Xia et al., 2020).

**FIGURE 1:**
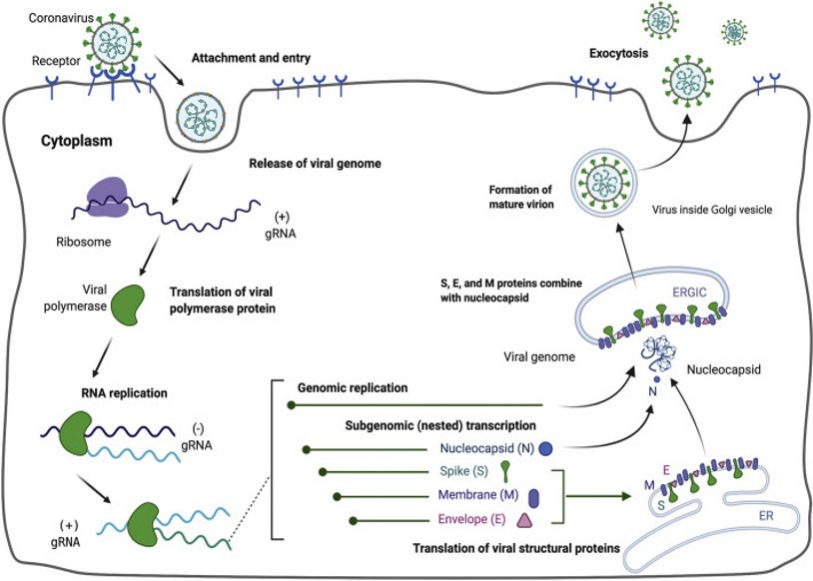
Depicts the mechanism of SARS‐CoV‐2 entry, replication, and maturation of the virion mediated through the attachment to the host cell

The coronavirus attachment to the host cell is initiated by the interaction of S‐protein with the host receptor through membrane fusion and endocytosis (Shang, Wan, Luo, et al., 2020; Walls et al., 2020). The S‐protein comprises S1 receptor‐binding subunit possessing the S1‐N‐terminal domain (NTD) (15–300 aa) and S1‐C‐Terminal domain (CTD) (326–567 aa), and the S2 membrane fusion domain that is involved in aiding viral RNA entry into the host cell post S1‐RBD‐ACE‐2 interaction. In particular, S1‐CTD serves as the RBD for all human coronaviruses. Receptor binding induces structural changes in the S‐protein that comprises S1 and S2 domains, which eventually splices the two domains with the help of protease. Post receptor binding, acid‐dependent proteolytic cleavage of S‐protein occurs mediated by protease enzymes, along with the fusion of S2 domain that assists in the release of viral RNA into the host cell. Then, the viral replisome complexes are assembled, followed by the synthesis of genomic and sub‐genomic RNAs, which are produced through negative‐strand intermediates, and from which the sub‐genomic RNA integrates with the host RNA and undergoes nested transcription (Shang, Wan, Luo, et al., 2020). The spike, membrane, and envelope structural proteins are first translated through the host‐viral mRNA machinery and are exported to the host endoplasmic reticulum (ER) that translocate along a secretory pathway into the endoplasmic reticulum–Golgi intermediate compartment (ERGIC) (Shang, Wan, Luo, et al., 2020; Tai et al., 2020; Walls et al., 2020; Xia et al., 2020). Then, the viral nucleocapsids bud into the ERGIC membranes that comprise viral structural proteins, thereby producing mature virions (Figure 1). Then, the virions are transported by the vesicles to the cell surface and are released by exocytosis.

The cornerstone for controlling the virus might be effective vaccines or drugs that are still under research pursuit, and due to which almost a third of the global population is affected by the virus. Studies aimed at finding key drug compounds or vaccine candidates targeting the S‐protein RBD‐ACE‐2 complex have been under pursuit for prompt treatment and research purpose. As there is no proper treatment for SARS‐CoV‐2 at present, it is highly crucial to search for direct therapeutics as well as natural therapeutics or immune boosters or virucidal products that can assist in containing the spread of the virus. Natural substances from herbs or mushrooms have been shown to possess potent antiviral properties that can be explored as therapeutics for SARS‐CoV‐2 (Shahidi and Camargo, 2021; Mohiuddin, 2021; Shahzad et al., 2020; Pan et al., 2013). In this regard, Chaga (*Inonotus obliquus*), a traditional edible mushroom, is well known for its therapeutic value. Chaga mushroom polysaccharides have been found in several studies to possess biologically active substances, in particular long chain homopolysaccharide beta‐glucan, galactomannan, and the unique terpenoid betulinic acid (Gao et al., 2005; Kim, 2005; Chen & Wang, 2014; Glamočlija et al., 2015; Szychowski et al., 2020; Peng and Shahidi, 2020; Lu et al., 2021; Basal et al., 2021). The virucidal activity of crude Chaga extract was previously proved against feline coronavirus (FCoV) (Tian et al., 2017) and hepatitis virus (Pan et al., 2013), suggesting a promising potential application in developing antiviral regimens against the current novel pandemic due to SARS‐CoV‐2. We hence attempted to target the S‐protein of SARS‐CoV‐2 with the unique components of Chaga mushroom for developing a potent natural therapeutic that can perform dual roles: aid in inhibiting the viral entry by interacting with the specific viral S‐protein interacting sites involved in ACE‐2‐RBD interaction, and boosting the immunity and reducing inflammation in the hosts, thereby assisting in preventing the cytokine storm and sudden inflammatory spurt that is responsible for maximum fatalities.

## 2 METHODS

### 2.1 Spike protein amino acid sequence retrieval and phylogenetic analysis

Global SARS‐CoV‐2 S‐protein amino acid sequences (*n* = 202) were retrieved from Uniprot and comprised a set of sequences from bat, pangolin, and human samples involved in previous and recent outbreaks. Multiple sequence alignment (MSA) was performed using Clustal W program embedded in Mega software (Figure S1). Maximum‐likelihood method and generalized time‐reversible model of amino acid substitution were utilized to perform the phylogenetic analysis in Mega 5 software (Tamura et al., 2011). Bootstrap replications of 1,000 values of the Nearest‐Neighbor Interchange procedure were used to estimate the robustness of each node, along with estimating the genetic distance using the maximum likelihood method.

### 2.2 Preparation of receptor protein

The cryo‐electron microscopy structure of SARS‐CoV‐2 protein (PDB ID: 6VSB) was chosen for initial structure preparation as it is explicitly demonstrated with its ligand interacting sites (Wrapp et al., 2020). First, all the water molecules and heteroatoms were removed in Autodock tool. Then, hydrogen atoms were added to the model based on an explicit all atom model. Finally, Kollmann charges were added for ensuing interaction with the ligands, and the model was energy minimized.

### 2.3 Ligand preparation

**FIGURE 2:**
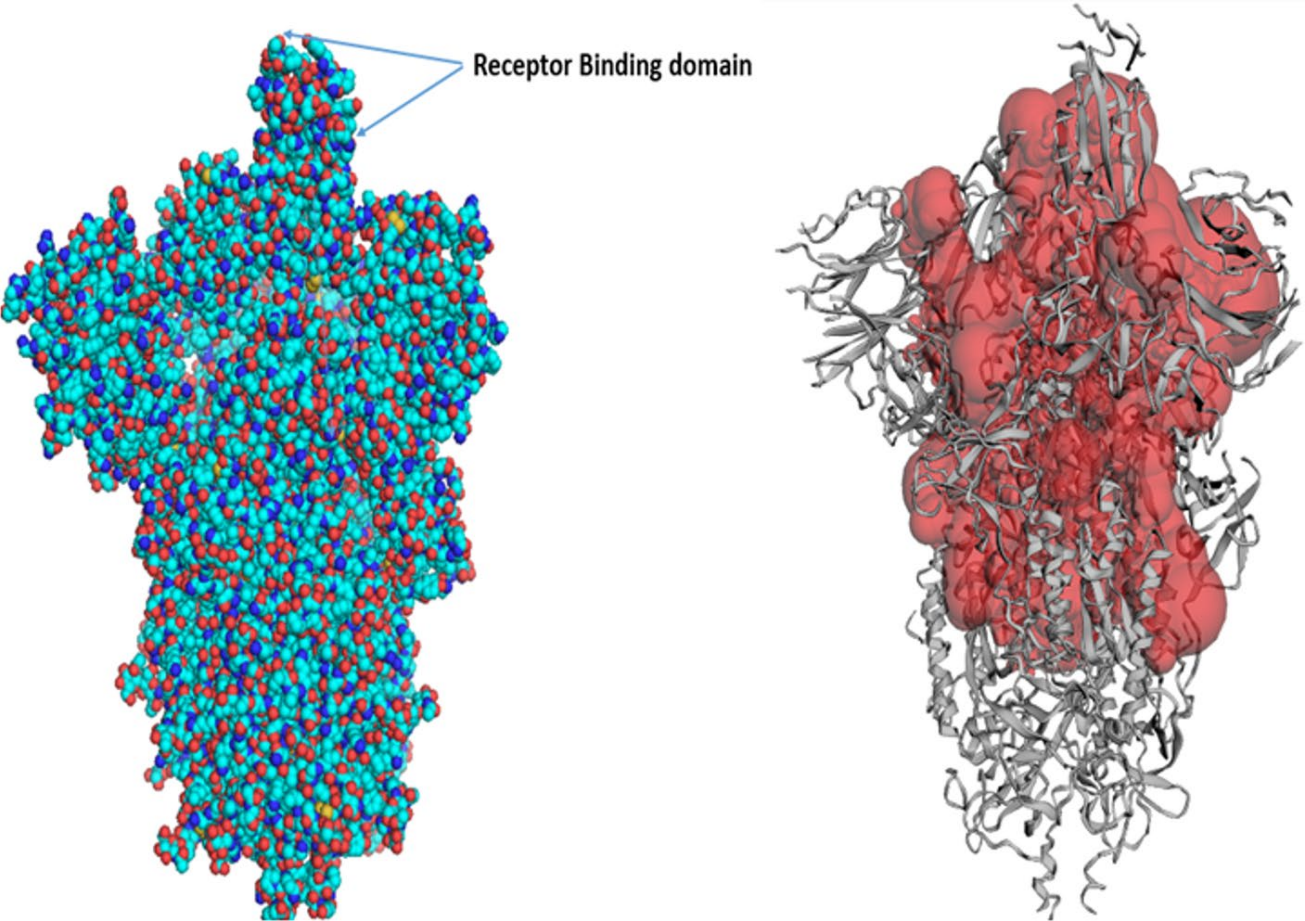
The compact structure of spike glycoprotein showing the receptor‐binding domain of SARS corona virus (COVID‐19). Right. Active site pockets (red) of S‐glycoprotein from CastP server based on maximum surface area (33,706.38) and surface volume (63,378.53) available for ligand binding

The 3D conformers of Chaga mushroom components beta glycan (CID: 439262), galactomannan (CID 439336), and betulinic acid (CID: 64971) were retrieved in the 3D conformer state from Pubchem (Figure 2) and were prepared for docking in the Ligand Preparation tool of Discovery Studio v20.1.0.19295 (Studio, 2008). In addition, 3D conformer NAG (2‐Acetamido‐2‐deoxy‐3‐O‐beta‐D‐glucopyranuronosyl‐beta‐D‐glucopyranose; CID: 5288907) that has potent binding affinity for the S‐protein of SARS‐CoV‐2 was also retrieved from Pubchem for comparison purposes and prepared as described above. The root was detected for each ligand, which were eventually saved in.pdbqt files similar to that of receptor protein.

### 2.4 Molecular docking and post scoring analysis

**FIGURE 3:**
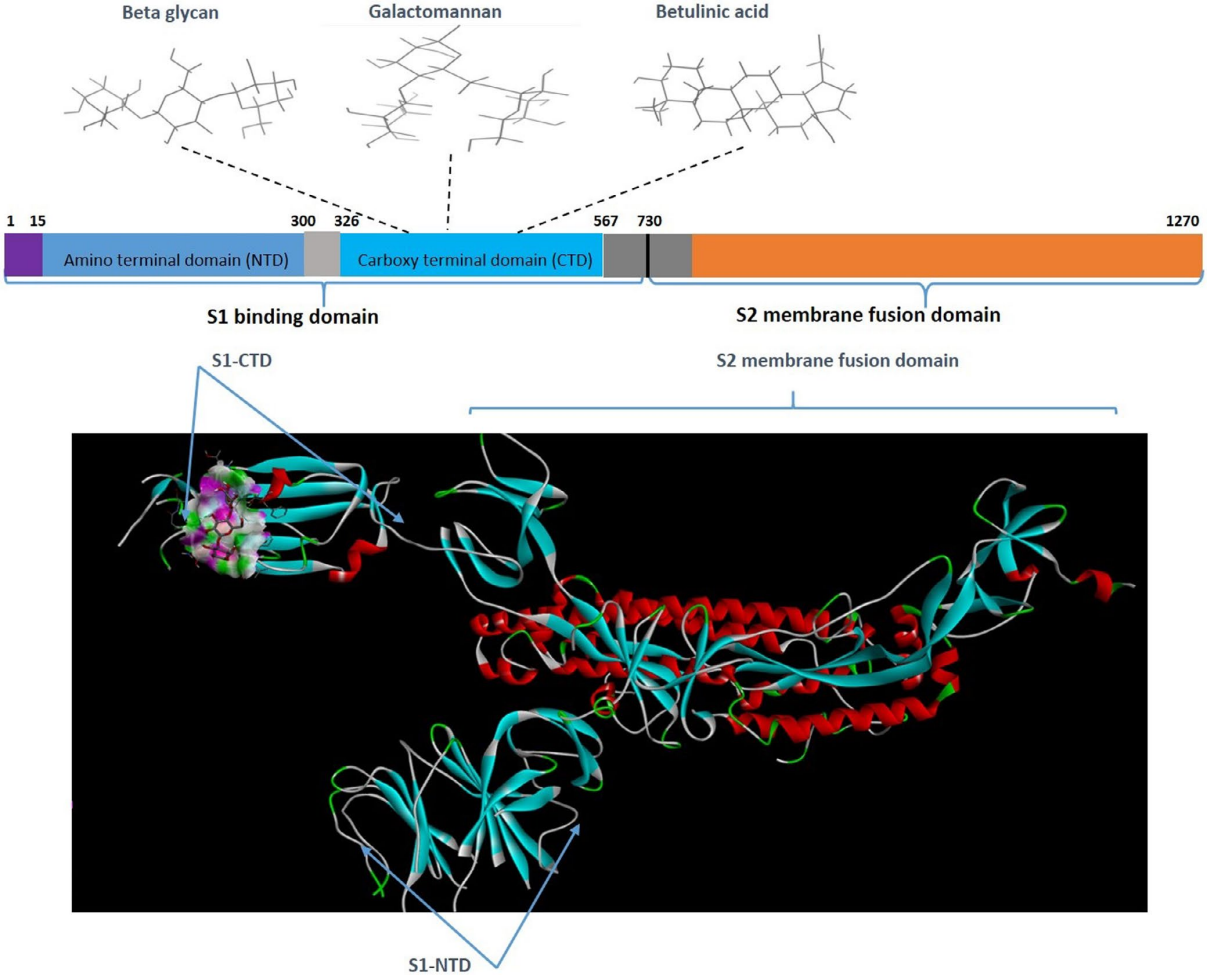
Above. The architecture of S‐protein showing the different types of domains involved in the host virus interaction and virus entry. All three natural ligands interacted with the S1‐CTD of the spike protein, indicating their potential to specifically bind to the RBD. Below: 3D cartoon model of chain A of spike protein of corona virus showing the S1 and S2 domains and the specific interaction region (S1 carboxy‐terminal domain, CTD) of the chaga mushroom components

SARS‐CoV‐2 cryo‐electron microscopic structure (PDB ID: 6VSB) was employed for the docking analysis. Because SARS‐CoV‐2 is a homotrimer consisting of chain A, B, and C, chain A of the spike protein was used for the docking experiment. The active site of the target protein was predicted using the binding site module of Discovery studio (Studio, 2008) and CastP server (Tian et al., 2018) based on maximum volume (Figure 3). Blind docking with the complete spike protein was performed using Autodock tools to predict the overall receptor–ligand interactions. First, both the receptor spike protein and the ligands were processed in Autodock tools as described above and converted to.pdbqt format. Autodock Vina (Trott & Olson, 2010) docking tool was used to assess the binding ability of chaga mushroom components and viral S‐protein. Blind docking was performed to know the probable binding sites on the spike protein. For this, the entire S‐protein was selected with a grid box of dimension 75 Å × 96 Å × 159 Å with grid spacing 1 Å. The receptor protein was kept rigid whereas the ligands were flexible. Eight sets of docking poses were performed with exhaustiveness 100 in Autodock Vina; each set produced 11 conformations. Among them, 9 conformations consistently docked at the RBD domain of S‐protein, which was again used for the local docking/site‐specific docking. For site‐specific docking, S‐protein of SARS‐CoV‐2 was selected with a grid box of dimension 58 Å × 55 Å × 65 Å (corresponding to the RBD site) and a grid spacing of 1 Å and exhaustiveness at 100. Six sets of site‐specific docking poses were analyzed for each ligand, and the site exhibiting the maximum number of binding conformations was indicated as a potential interacting site, followed by the assessment of hydrophilic and hydrophobic interactions in Pymol software. The interacting sites were further visualized in Discovery Studio Visualizer, and the 2D interaction, nature, types of bonds, and bond lengths were determined. The ligand–protein structures were also analyzed for molecular dynamics simulation mode of the ligand molecules docked with the protein using GROMACS 2018.3. Protein configuration file topology was generated using the GROMOS96 43a1 force field. Ligand topology was predicted through PRODRG2 server and the whole analysis was set for a MD simulation run for 100 ns time interval in GROMACS package, followed by visualization of the structure by PyMOL. Finally, rescoring of the docked conformations was performed in Mol Dock program for better clarity and refinement of ligand–protein interactions.

## 3 RESULTS AND DISCUSSION

### 3.1 Phylogenetic analysis of spike protein

The evolutionary analysis revealed an unprecedented mode of SARS‐CoV‐2 evolution and transmission across humans, particularly in the recent pandemic. Phylogenetic analysis of the S‐protein of SARS‐CoV‐2 sequences (*n* = 202) retrieved across the globe from GenBank was represented by a ML tree showing unique pattern of SARS‐CoV‐2 lineages (Figure S2). The analysis revealed that SARS‐CoV‐2 specifically originated in bats (outgroup), followed by intermittent transmission through pangolins, and then to humans. The human samples across the globe are grouped into a single major cluster, with several minor subgroups emerging depending on the geographical regions with high similarity indices. Amino acid multiple sequence alignment showed high conservativeness in the amino acid residues across all the samples. However, there was unique conservativeness in the amino acids 680–686 in all the recent COVID‐19 samples compared with pre‐2019 viral sequences, thereby suggesting its advantageous role in virus evolution and pathogenesis (highlighted in yellow, Figure S2). This revealed that the recent SARS‐CoV‐2 virus possesses a furin cleavage site near the S1/S2 junction from 680 to 686 bp (NSPRRA), which was absent in the previous virus. Furin cleavage site can cause the excision of the two segments by the furin enzyme which eventually assists in gaining entry into the host through precise fusion and proteolysis (Walls et al., 2020). This was a key difference in the SARS outbreaks from previous and current outbreaks and indicated that the RBD is being selectively evolving. Besides, several residues in the S1‐CTD domain that have been reported to be key residues for binding of SARS‐CoV‐2 to human ACE‐2 such as ASN‐343, TRP‐436, ASN‐437, ASN‐440, TYR‐442, LEU‐472, ASN‐473, TYR‐475, ASN‐479, TYR‐484, THR‐486, and THR‐487 (Chen et al., 2020; Shang, Wan, Liu, et al., 2020) were found to be positively selected and conserved in our phylogenetic analysis. Therefore, drugs/small molecules targeting these regions could play key roles in modulating the viral entry into the host cells.

### 3.2 Molecular docking analysis

**FIGURE 4:**
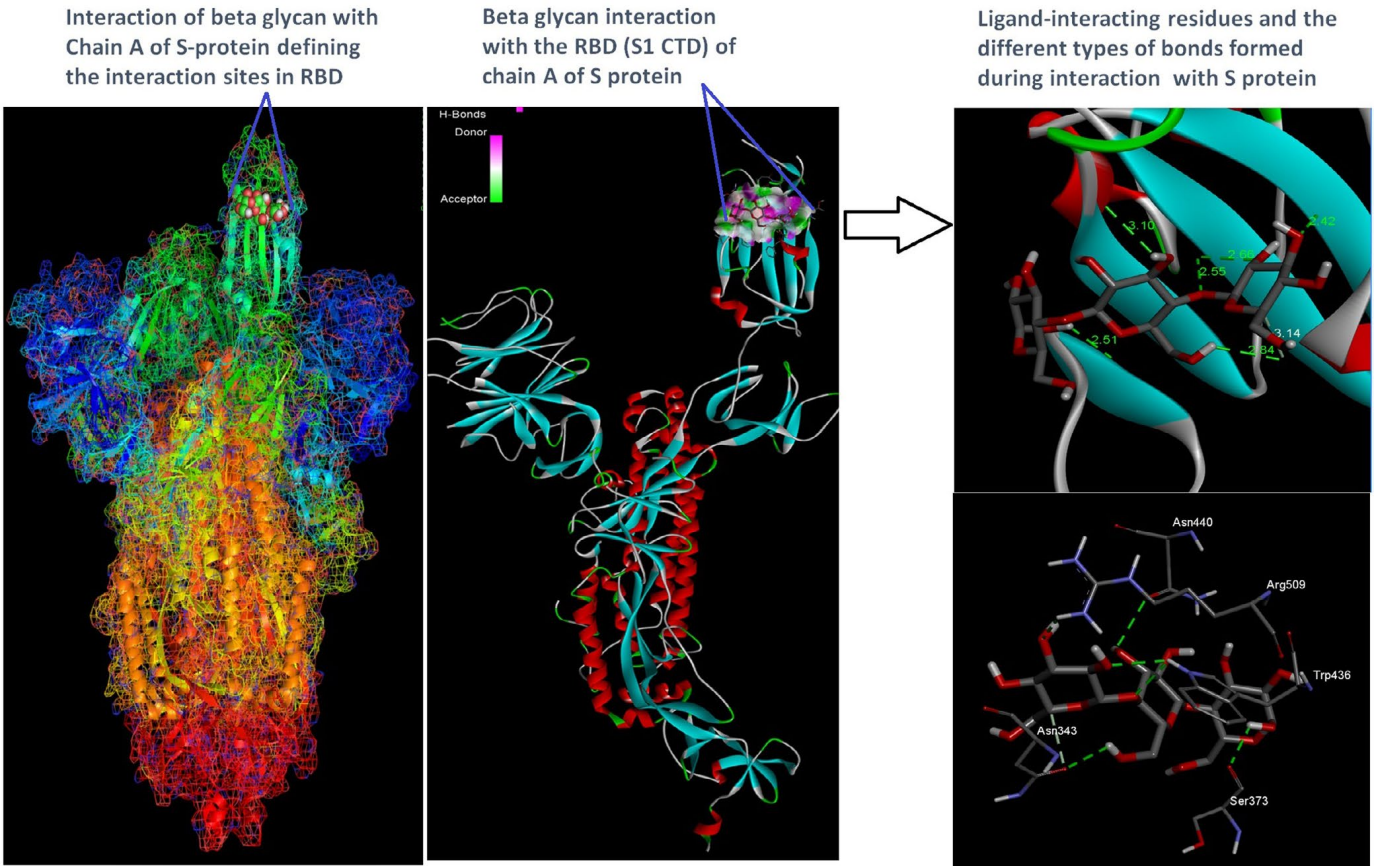
Beta‐glucan ligand and spike protein docking results showing the ligand interaction site, 2D interactions, residues involved in maximum interaction, type of bonds, and bond lengths formed for depicting the overall analysis

**FIGURE 5:**
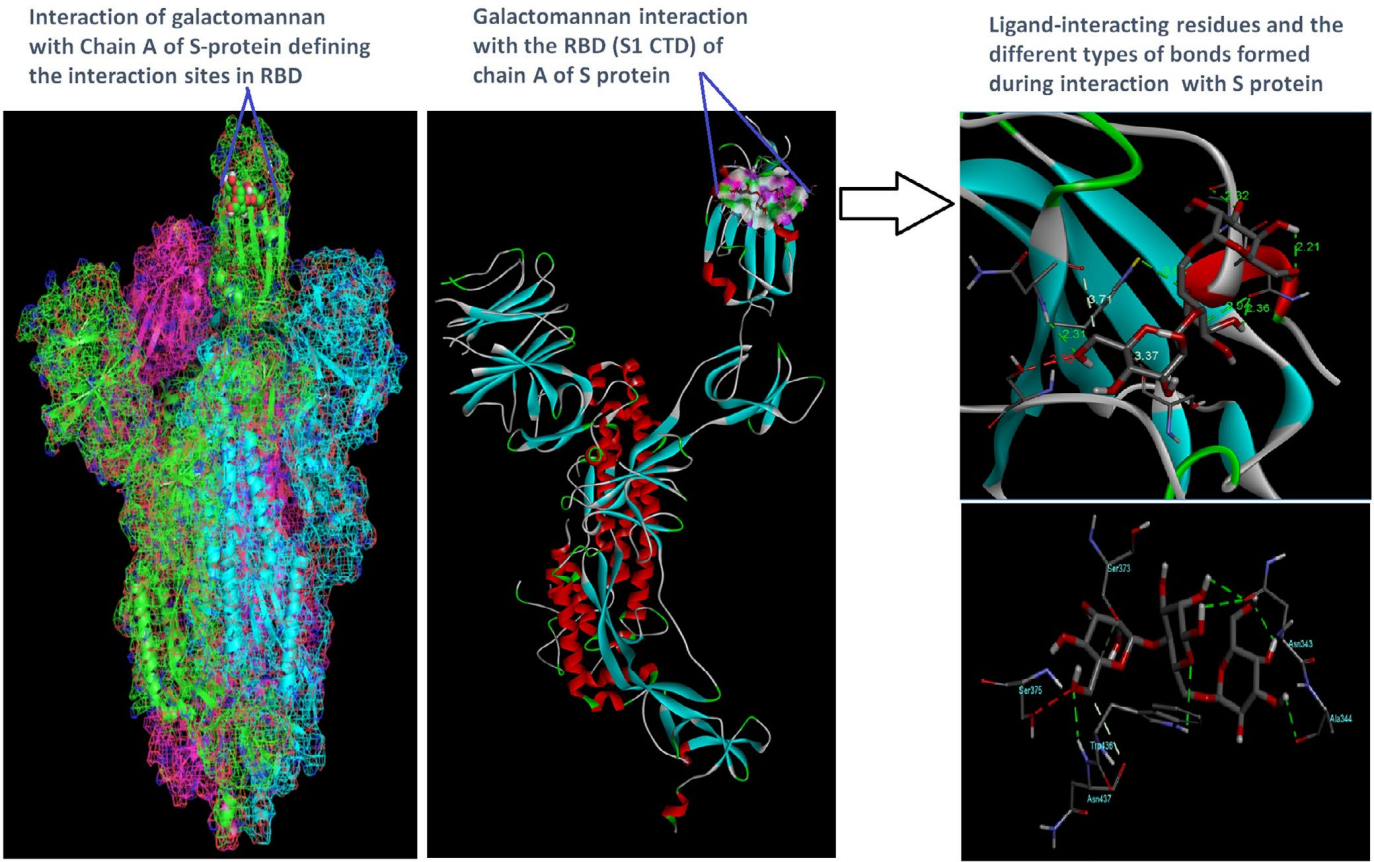
Galactomannan ligand and spike protein docking results showing the ligand interaction site, 2D interactions, residues involved in maximum interaction, type of bonds, and bond lengths formed for depicting the overall analysis

**FIGURE 6:**
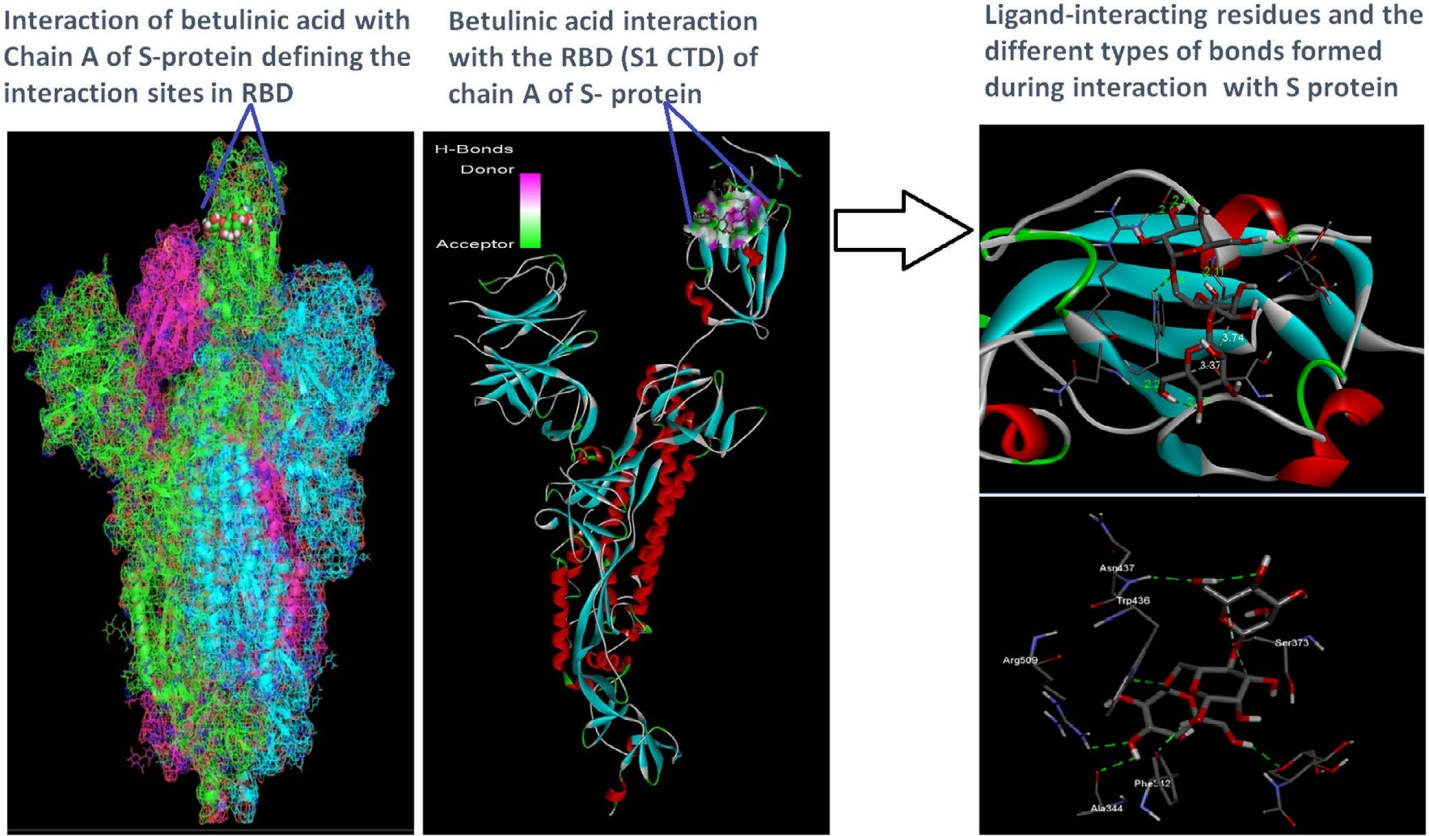
Betulinic acid ligand and spike protein docking results showing the ligand interaction site, 2D interactions, residues involved in maximum interaction, type of bonds, and bond lengths formed for depicting the overall analysis

**FIGURE 7:**
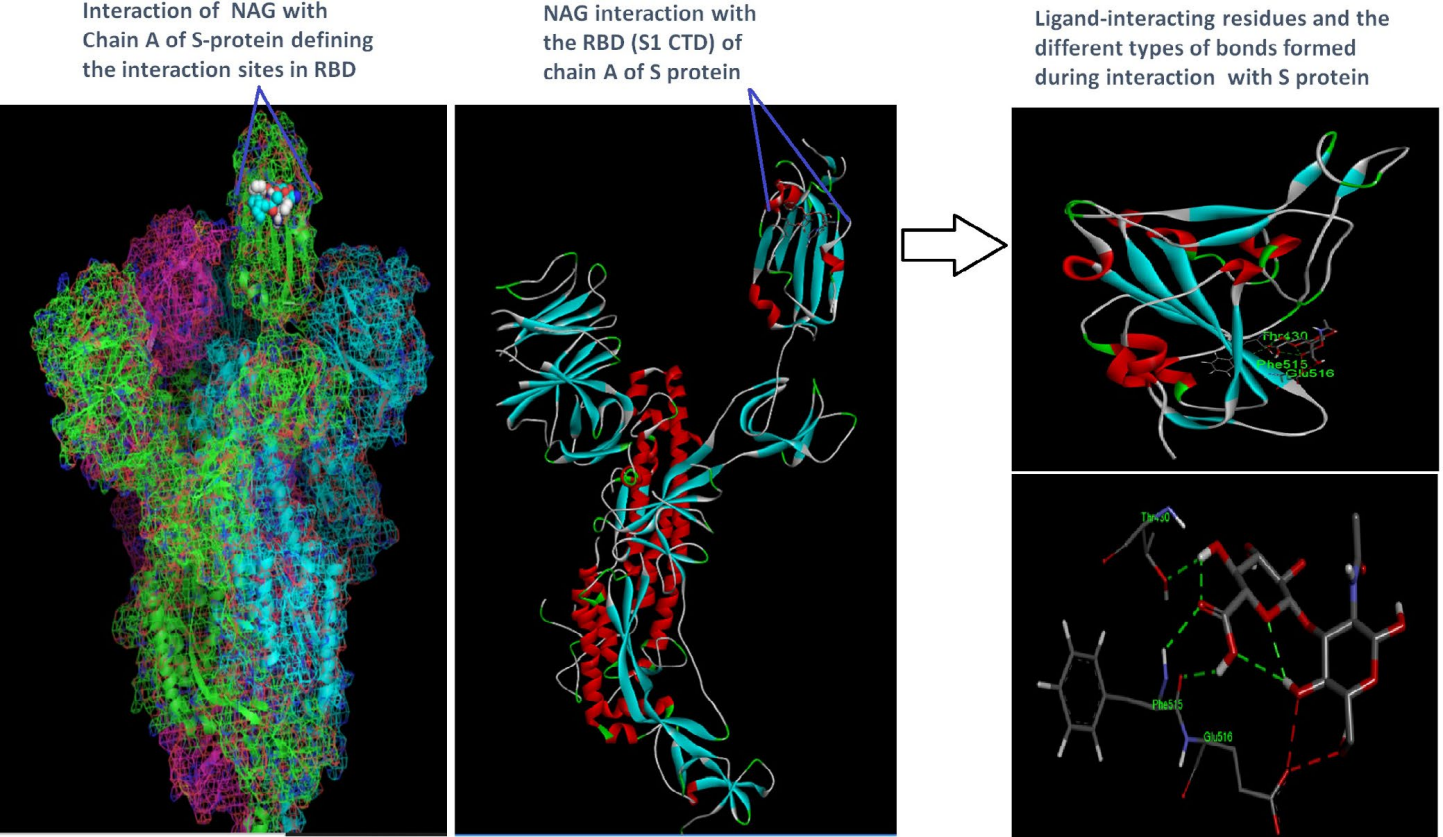
Control molecule NAG (2‐acetamido‐2‐deoxy‐beta‐D‐glucopyranose) and spike protein docking results showing the ligand interaction site, 2D interactions, residues involved in maximum interaction, type of bonds, and bond lengths formed for depicting the overall analysis

Docking analysis revealed that Chaga mushroom components (beta glycan, galactomannan, and betulinic acid) bound to the S1‐CTD residues of SARS‐CoV‐2 (PDB: 6VSB) involved in ACE‐2‐RBD interaction (Figures 4, 5, 6, 7). Computational assessment using Autodock tools for S‐protein and the three ligands beta glycan, galactomannan, and betulinic acid revealed that the highest interaction occurred at the RBD involving site 2 (*X* = 212.255, *Y* = 194.938, *Z* = 285.952). The Autodock and Moldock score values among the ligands varied between −7.40 kcal/mol and −8.67 kcal/mol and −24.71 kcal/mol and −32.78 kcal/mol respectively. For beta glycan, the best interacting site comprised the amino acids: ASN‐440, SER‐373, TRP‐436, ASN‐343, and ARG‐509 for the protein–ligand interaction with minimum energy required for stability (energy minimization, −8.4 kcal). The binding site cavity showed the presence of all the active amino acid residues. For the interaction of 6VSB and galactomannan, the best interacting site was the same site 2 and comprised amino acids ASN‐437, SER 373, TRP‐436, ASN‐343, and ALA 344 that interacted with the receptor S‐glycoprotein with high binding energy of −7.4 kcal. For the interaction of 6VSB and betulinic acid, the best interacting site was the same site 2 and comprised amino acids ASN‐437, SER‐373, TRP‐436, PHE‐342, ARG‐509, and ALA‐344 that interacted with the S‐protein with a high binding energy of −8.1 kcal. Further analysis showed that the TRP‐436, followed by ASN‐440, was detected to be the amino acids having maximum affinity to all the three ligands and was shown to be hydrogen bonded strongly with the receptor protein. The control molecule NAG demonstrated a binding energy of ΔG = −8.6 Kcal/mol with the interacting residues, such as THR 430, PHE 515, and GLU 516 that resided near the active site region and formed 4 hydrogen bonds. The RMSD values for all the above ligand–protein conformations ranged between 0.6 and 0.8 nm till 100 ns during MD simulation, which demonstrated high stability of the complexes. Furthermore, the amino acids in the active site residues formed H bonds with the ligand molecule and remain stable throughout the time frame. Therefore, the molecular interaction results in this study revealed that all the ligand interacting sites were located in the S1 domain (highlighted in yellow) and can be thus considered as crucial targets for SARS‐CoV‐2 spike protein. The interacting sites were primarily hydrophilic in nature and suggested strong interaction with the ligands. A positive correlation was obtained between the interaction of S‐protein and galactomannan, betulinic acid, and beta glycan in an ascending order of binding energies (−7.4 > −8.1 > −8.4 kcal/mol). The conformations with maximum negative binding energy are represented in the figures, and different types of interactions and the nature and types of bonds are shown in Table 1.

**TABLE 1:** Amino acid residues of 6VSB.pdb involved in interaction with the chaga mushroom components: beta glycan, galactomannan, betulinic acid, and control molecule NAG

Beta‐Glucan (CID: 439262)						
Residues	Donor	Acceptor	Bond type	Binding energy (kcal/mol.)	Vina score (kcal/mol)	Moldock score (Kcal/mol)
TRP‐436	HE1	O	Conventional hydrogen bond	−8.40	−0.8.4	−32.78
ASN‐440	H	OD1	Conventional hydrogen bond	−8.47		
ASN‐343	H, C	OD1	Conventional hydrogen bond, Carbon hydrogen bond	‐−8.49		
ARG 509	H22	O	Conventional hydrogen bond	−8.71		
SER 373	H	O	Conventional hydrogen bond	−9.01		
Galactomannan (CID 439336)						
TRP‐436	HE1	O2	Conventional hydrogen bond, single	−7.40	−7.4	−24.4
ASN‐437	HN	O15	Conventional hydrogen bond	−7.42		
ASN‐343	H57, H63	OD1	Conventional hydrogen bond, double	−7.42		
ALA 344	H63	O	Conventional hydrogen bond	−7.49		
SER 373	C27	O	Carbon hydrogen bond	−7.9		
Betulinic acid (CID: 64971)						
TRP‐436	HE1	O4	Conventional hydrogen bond	−8.21	−8.1	−25.3
ASN‐437	HN	O15	Conventional hydrogen bond	−8.13		
PHE 342	H61	O	Conventional hydrogen bond	−7.98		
ALA 344	H63	O	Conventional hydrogen bond	−7.91		
ARG 509	H22	O13	Carbon hydrogen bond	−7.95		
SER 373	C19	O	Conventional hydrogen bond	−7.96		
NAG (CID: 5288907)						
THR 430	HN	O2	Conventional hydrogen bond	−8.8	−8.67	−31.3
PHE 515	H1	O15	Conventional hydrogen bond	−8.8		
PHE 515	H1	O	Conventional hydrogen bond	−8.6		

**FIGURE 8:**
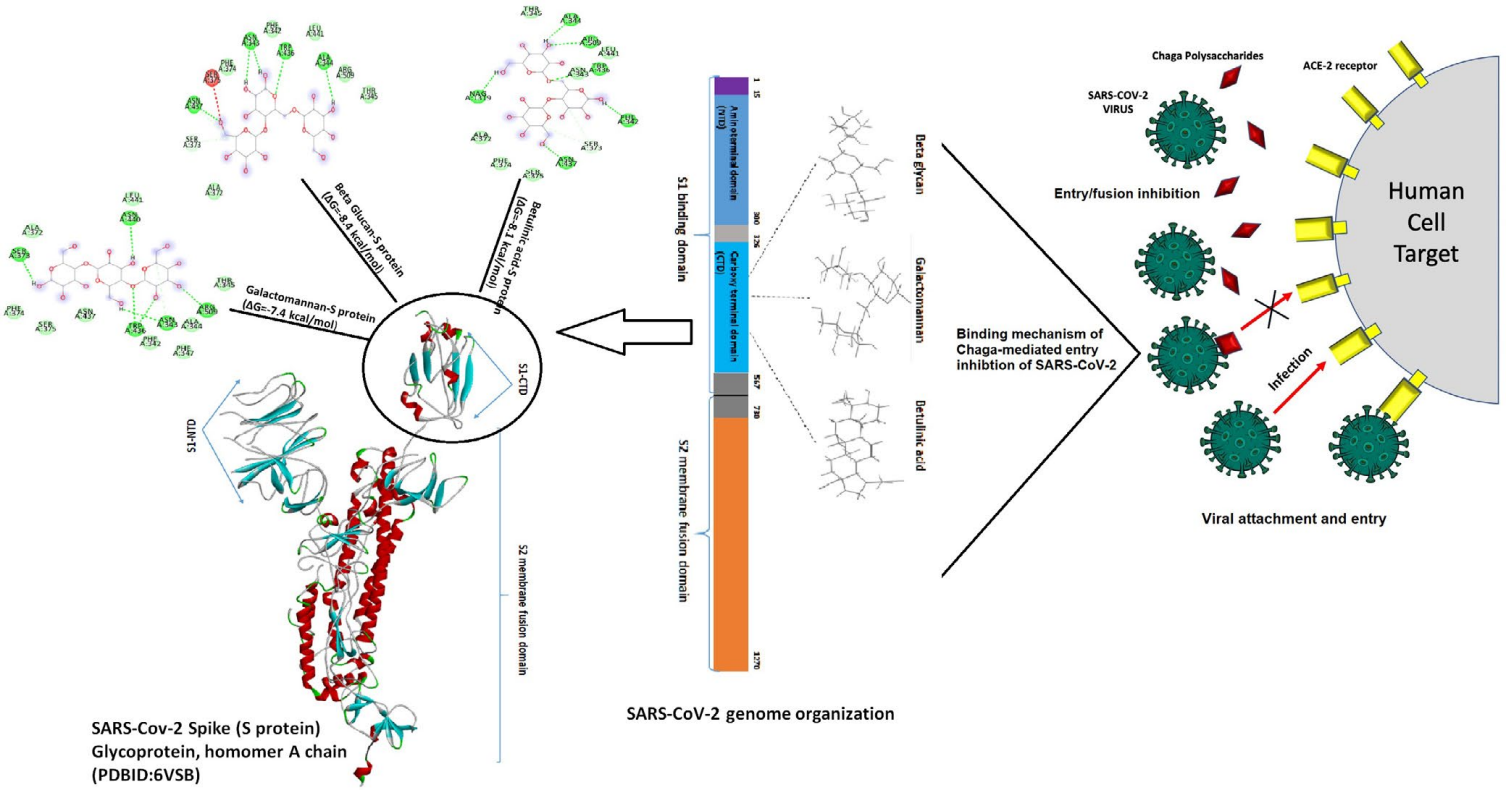
Mechanistic diagram depicting the overall interaction of Chaga mushroom components beta glycan, galactomannan, and betulinic acid with the spike protein (S‐protein) of SARS‐CoV‐2. The three Chaga components specifically interacted with the S1‐carboxy‐terminal domain of the SARS‐CoV‐2 with relatively high binding energies, which could potentially modulate the S‐protein and implicate in inhibition of virus entry into the host cell

Through docking, we could gain insight into the interactions of the ligands with the amino acids in the binding pocket of the virus S‐protein. Docking results demonstrated significant binding interaction of the S1‐RBD of the SARS‐CoV‐2 with beta glycan, galactomannan, and betulinic acid. More importantly, all the ligand interacting sites specifically fitted within the carboxy‐terminal domain of the RBD (Figure 8) and included amino acid residues that have been previously reported to aid the virus in host entry in a conservative manner, such as TRP‐436, ASN‐437, and ASN‐440. Such interaction of Chaga mushroom components with the S‐protein of SARS‐CoV‐2 could modulate its binding and thus can cause inhibition of virus entry into the host cell. Besides, the docking results were at par with the binding affinity of control molecule NAG with S‐protein that has been reported to exhibit strong binding affinity for S‐protein (Tai et al., 2020). Therefore, Chaga mushroom would also exhibit similar effects by various interactions and modulate the virus–host cell interaction.

The versatile benefits of Chaga mushroom could be attributed to its unique composition through which it can specifically targeting the S1‐RBD of SARS‐CoV‐2 (Ibrahim et al., 2021). Chaga mushroom is a natural compound and indicates no side effects when administered in proper dosages (Eid et al., 2021). It also enhances specific innate immunity system and assists in lowering the proinflammatory cytokines, such as IL‐6, IL‐10, TNF‐α, MCp, and many others. (Van et al., 2009; Mishra et al., 2012; Balandaykin & Zmitrovich, 2015). This attribute will greatly assist in reducing the case fatalities of SARS‐CoV‐2 due to a phenomenon called “cytokine storm” that results in an uncontrollable amplification and recruitment of inflammatory cytokines and immune cells to combat the infection, eventually resulting in organ damage and death (Pedersen & Ho, 2020). Therefore, Chaga mushroom can be a health‐promoting booster in severe SARS‐CoV‐2 cases that exhibit excessive inflammation. Hence, delineating the role of Chaga mushroom components in SARS‐CoV‐2 interaction using and laboratory‐based studies and clinical trials could reveal promising potential for the development of natural antiviral therapeutics.

## 4 CONCLUSION

Chaga mushroom components, beta glycan, galactomannan, and betulinic acid exhibited strong binding interaction with the S1‐carboxy‐terminal domain of the receptor‐binding domain of SARS‐CoV‐2, mainly at TRP‐436, ASN‐437, and ASN‐440 sites. Chaga mushroom shows promise in interacting with the viral spike protein and can be further explored in clinical settings that can bolster the current treatment regime for SARS‐CoV‐2. This will assist in developing natural anti‐coronavirus therapeutics in future that can greatly supplement the use of current anti‐SARS‐CoV‐2 drugs.

## CONFLICTS OF INTEREST

The authors declare that they do not have any conflict of interest.

## AUTHOR CONTRIBUTIONS

**Jehane Eid:** Conceptualization (equal); Data curation (equal); Formal analysis (equal); Funding acquisition (equal); Methodology (equal); Resources (equal); Software (equal); Supervision (equal); Visualization (equal); Writing – original draft (equal); Writing – review and editing (equal). **Biswadeep Das:** Conceptualization (equal); Data curation (equal); Formal analysis (equal); Methodology (equal); Software (equal); Supervision (equal); Validation (equal); Visualization (equal); Writing – original draft (equal); Writing – review and editing (equal). **Majdah Al – Tuwaijri:** Formal analysis (equal); Visualization (equal); Writing – review and editing (equal). **Wesam Basal:** Data curation (equal); Formal analysis (equal); Visualization (equal); Writing – original draft (equal).

## ETHICAL APPROVAL

This study does not involve any human or animal testing.

## Supporting information

Fig S1

Fig S2

## Data Availability

Data available in article supplementary material.

## References

[fsn32576-bib-0001] Balandaykin, M. E. , & Zmitrovich, I. V. (2015). Review on chaga medicinal mushroom, inonotus obliquus (Higher Basidiomycetes): Realm of medicinal applications and approaches on estimating its resource potential. International Journal of Medicinal Mushrooms, 17(2), 95–104. 10.1615/IntJMedMushrooms.v17.i2.10 25746615

[fsn32576-bib-0002] Basal, W. T. , Elfiky, A. , & Eid, J. (2021). Chaga medicinal mushroom *Inonotus* *obliquus* (agaricomycetes) terpenoids may interfere with SARS‐CoV‐2 spike protein recognition of the host cell: A molecular docking study. International Journal of Medicinal Mushrooms, 23(3), 1–14. 10.1615/IntJMedMushrooms.2021037942 33822495

[fsn32576-bib-0003] Chen, H. , & Wang, J. (2014). Phytochemistry, traditional uses and health benefits of the mushroom inonotus obliquus (Chaga). In: Mushrooms Cultiv Antioxid Prop Health Benefits. p. 93‐118.

[fsn32576-bib-0004] Chen, Y. , Liu, Q. , & Guo, D. (2020). Emerging coronaviruses: Genome structure, replication, and pathogenesis. Journal of Medical Virology, 92(4), 418–423. 10.1002/jmv.25681 31967327 PMC7167049

[fsn32576-bib-0005] Eid, J. I. , Al‐Tuwaijri, M. M. , Mohanty, S. , & Das, B. (2021). Chaga mushroom (*Inonotus* *obliquus*) polysaccharides exhibit genoprotective effects in UVB‐exposed embryonic zebrafish (*Danio* *rerio*) through coordinated expression of DNA repair genes. Heliyon, 7(2), e06003. 10.1016/j.heliyon.2021.e06003 33598573 PMC7868817

[fsn32576-bib-0006] Gao, Y. , Tang, W. , Gao, H. E. , Chan, E. , Lan, J. , Li, X. , & Zhou, S. (2005). Antimicrobial activity of the medicinal mushroom Ganoderma. Food Reviews International, 21(2), 211–229. 10.1081/FRI-200051893

[fsn32576-bib-0007] Glamočlija, J. , Ćirić, A. , Nikolić, M. , Fernandes, Â. , Barros, L. , Calhelha, R. C. , Ferreira, I. C. F. R. , Soković, M. , & Van Griensven, L. J. L. D. (2015). Chemical characterization and biological activity of Chaga (Inonotus obliquus), a medicinal “mushroom”. Journal of Ethnopharmacology, 162, 323–332. 10.1016/j.jep.2014.12.069 25576897

[fsn32576-bib-0008] Ibrahim, I. M. , Elfiky, A. A. , & Elgohary, A. M. (2021). Recognition through GRP78 is enhanced in the UK, South African, and Brazilian variants of SARS‐CoV‐2; An in silico perspective. Biochemical and Biophysical Research Communications, 562, 89–93. 10.1016/j.bbrc.2021.05.058 34049205 PMC8139235

[fsn32576-bib-0009] Kim, Y.‐R. (2005). Immunomodulatory activity of the water extract from medicinal mushroom inonotus obliquus. Mycobiology, 33(3), 158–162. 10.4489/myco.2005.33.3.158 24049493 PMC3774877

[fsn32576-bib-0010] Lu, Y. , Jia, Y. , Xue, Z. , Li, N. , Liu, J. , & Chen, H. (2021). Recent developments in *Inonotus* *obliquus* (Chaga mushroom) polysaccharides: Isolation, structural characteristics, biological activities and application. Polymers, 13(9), 1441. 10.3390/polym13091441 33947037 PMC8124789

[fsn32576-bib-0011] Machhi, J. , Herskovitz, J. , Senan, A. M. , Dutta, D. , Nath, B. , Oleynikov, M. D. , Blomberg, W. R. , Meigs, D. D. , Hasan, M. , Patel, M. , Kline, P. , Chang, R.‐C. , Chang, L. , Gendelman, H. E. , & Kevadiya, B. D. (2020). The natural history, pathobiology, and clinical manifestations of SARS‐CoV‐2 infections. Journal of Neuroimmune Pharmacology: The Official Journal of the Society on NeuroImmune Pharmacology, 15(3), 359. 10.1007/s11481-020-09944-5 32696264 PMC7373339

[fsn32576-bib-0012] Mishra, S. K. , Kang, J.‐H. , Kim, D.‐K. , Oh, S. H. , & Kim, M. K. (2012). Orally administered aqueous extract of Inonotus obliquus ameliorates acute inflammation in dextran sulfate sodium (DSS)‐induced colitis in mice. Journal of Ethnopharmacology, 143(2), 524–532. 10.1016/j.jep.2012.07.008 22819687

[fsn32576-bib-0013] Mohiuddin, A. K. (2021). Can medicinal mushrooms fight against sars‐cov‐2/covid‐19? Journal of Internal Medicine: Science & Art, 2, 23–24. 10.36013/jimsa.v2i1.57

[fsn32576-bib-0014] Pan, H. H. , Yu, X. T. , Li, T. , Wu, H. L. , Jiao, C. W. , Cai, M. H. , Li, X. M. , Xie, Y. Z. , Wang, Y. , & Peng, T. (2013). Aqueous extract from a Chaga medicinal mushroom, Inonotus obliquus (higher Basidiomycetes), prevents herpes simplex virus entry through inhibition of viral‐induced membrane fusion. International Journal of Medicinal Mushrooms, 15(1), 29–38. 10.1615/intjmedmushr.v15.i1.40 23510282

[fsn32576-bib-0015] Pedersen, S. F. , & Ho, Y.‐C. (2020). SARS‐CoV‐2: A Storm is Raging. Journal of Clinical Investigation, 130(5), 2202–2205. 10.1172/JCI137647 PMC7190904 32217834

[fsn32576-bib-0016] Peng, H. , & Shahidi, F. (2020). Bioactive compounds and bioactive properties of Chaga (*Inonotus* *obliquus*) mushroom: A review. Journal of Food Bioactives, 12, 9–75. http://www.isnff‐jfb.com/index.php/JFB/article/view/191. 10.31665/JFB.2020.12245

[fsn32576-bib-0018] Shahidi, F. , & de Camargo, A. C. (2021). Trends in food bioactives in the COVID‐19 pandemic year–JFB Audience. Journal of Food Bioactives, 13, 9–11. 10.31665/JFB.2020.13254

[fsn32576-bib-0019] Shahzad, F. , Anderson, D. , & Najafzadeh, M. (2020). The antiviral, anti‐inflammatory effects of natural medicinal herbs and mushrooms and SARS‐CoV‐2 infection. Nutrients, 12(9), 2573. 10.3390/nu12092573 PMC7551890 32854262

[fsn32576-bib-0020] Shang, J. , Wan, Y. , Liu, C. , Yount, B. , Gully, K. , Yang, Y. , Auerbach, A. , Peng, G. , Baric, R. , & Li, F. (2020). Structure of mouse coronavirus spike protein complexed with receptor reveals mechanism for viral entry. PLoS Pathogens, 16(3), e1008392. 10.1371/journal.ppat.1008392 32150576 PMC7082060

[fsn32576-bib-0021] Shang, J. , Wan, Y. , Luo, C. , Ye, G. , Geng, Q. , Auerbach, A. , & Li, F. (2020). Cell entry mechanisms of SARS‐CoV‐2. Proceedings of the National Academy of Sciences, 117(21), 11727–11734. 10.1073/pnas.2003138117 PMC7260975 32376634

[fsn32576-bib-0022] Sharma, A. , Tiwari, S. , Deb, M. K. , & Marty, J. L. (2020). Severe Acute Respiratory Syndrome Coronavirus‐2 (SARS‐CoV‐2): A global pandemic and treatments strategies. International Journal of Antimicrobial Agents, 56(2), 106054. 10.1016/j.ijantimicag.2020.106054 32534188 PMC7286265

[fsn32576-bib-0023] Studio, D. (2008). Discovery Studio Life Science Modeling and Simulations.

[fsn32576-bib-0024] Szychowski, K. A. , Bartosz, S. , Tadeusz, P. , & Jan, G. (2020). *Inonotus* *obliquus*–from folk medicine to clinical use. Journal of Traditional and Complementary Medicine, 11(4), 293–302. 10.1016/j.jtcme.2020.08.003 34195023 PMC8240111

[fsn32576-bib-0025] Tai, W. , He, L. , Pu, Z. X. , Voronin, D. , Jiang, S. , Zhou, Y. , & Du, L. (2020). Characterization of the receptor‐binding domain (RBD) of 2019 novel coronavirus: Implication for development of RBD protein as a viral attachment inhibitor and vaccine. Cellular & Molecular Immunology, 17, 613–620. 10.1093/nar/gky473 32203189 PMC7091888

[fsn32576-bib-0026] Tamura, K. , Peterson, D. , Peterson, N. , Stecher, G. , Nei, M. , & Kumar, S. (2011). MEGA5: Molecular evolutionary genetics analysis using maximum likelihood, evolutionary distance, and maximum parsimony methods. Molecular Biology and Evolution, 28(10), 2731–2739. 10.1093/molbev/msr121 21546353 PMC3203626

[fsn32576-bib-0027] Tian, J. , Hu, X. , Liu, D. , Wu, H. , & Qu, L. (2017). Identification of Inonotus obliquus polysaccharide with broad‐spectrum antiviral activity against multi‐feline viruses. International Journal of Biological Macromolecules, 95, 160–167. 10.1016/j.ijbiomac.2016.11.054 27865960 PMC7185483

[fsn32576-bib-0028] Tian, W. , Chen, C. , Lei, X. , Zhao, J. , & Liang, J. (2018). CASTp 3.0: Computed atlas of surface topography of proteins. Nucleic Acids Research, 46(W1), W363–W367. 10.1093/nar/gky473 29860391 PMC6031066

[fsn32576-bib-0029] Trott, O. , & Olson, A. J. (2010). Autodock vina: Improving the speed and accuracy of docking with a new scoring function, efficient optimization and multithreading. Journal of Computational Chemistry, 31(2), 455–461. 10.1002/jcc.21334 19499576 PMC3041641

[fsn32576-bib-0030] Van, Q. , Nayak, B. N. , Reimer, M. , Jones, P. J. H. , Fulcher, R. G. , & Rempel, C. B. (2009). Anti‐inflammatory effect of Inonotus obliquus, Polygala senega L., and Viburnum trilobum in a cell screening assay. Journal of Ethnopharmacology, 125, 487–493. 10.1016/j.jep.2009.06.026 19577624

[fsn32576-bib-0031] Walls, A. C. , Park, Y. J. , Tortorici, M. A. , Wall, A. , McGuire, A. T. , & Veesler, D. (2020). Structure, Function, and Antigenicity of the SARS‐CoV‐2 Spike Glycoprotein. Cell, 181(2), 281–292.e6. 10.1016/j.cell.2020.02.058 32155444 PMC7102599

[fsn32576-bib-0032] Wang, Q. , Zhang, Y. , Wu, L. , Niu, S. , Song, C. , Zhang, Z. , Lu, G. , Qiao, C. , Hu, Y. U. , Yuen, K.‐Y. , Wang, Q. , Zhou, H. , Yan, J. , & Qi, J. (2020). Structural and functional basis of SARS‐CoV‐2 entry by using human ACE2. Cell, 181(4), 894–904. 10.1016/j.cell.2020.03.045 32275855 PMC7144619

[fsn32576-bib-0033] WHO (2020). Coronavirus disease 2019 (COVID‐19) Situation Report – 183. https://www.who.int/docs/default‐source/wha‐70‐and‐phe/20200721‐covid‐19‐sitrep‐183.pdf?sfvrsn=b3869b3_2

[fsn32576-bib-0034] Wrapp, D. , Wang, N. , Corbett, K. S. , Goldsmith, J. A. , Hsieh, C. L. , Abiona, O. , Graham, B. S. , & McLellan, J. S. (2020). Cryo‐EM structure of the 2019‐nCoV spike in the prefusion conformation. Science, 367(6483), 1260–1263. 10.1126/science.aax0902 32075877 PMC7164637

[fsn32576-bib-0035] Xia, S. , Liu, M. , Wang, C. , Xu, W. , Lan, Q. , Feng, S. , Qi, F. , Bao, L. , Du, L. , Liu, S. , Qin, C. , Sun, F. , Shi, Z. , Zhu, Y. , Jiang, S. , & Lu, L. U. (2020). Inhibition of SARS‐CoV‐2 (previously 2019‐nCoV) infection by a highly potent pan‐coronavirus fusion inhibitor targeting its spike protein that harbors a high capacity to mediate membrane fusion. Cell Research, 30, 343–355. 10.1038/s41422-020-0305-x 32231345 PMC7104723

[fsn32576-bib-0036] Yan, R. , Zhang, Y. , Li, Y. , Xia, L. , Guo, Y. , & Zhou, Q. (2020). Structural basis for the recognition of SARS‐CoV‐2 by full‐length human ACE2. Science, 367(6485), 1444–1448. 10.1126/science.abb2762 32132184 PMC7164635

[fsn32576-bib-0037] Zimmermann, P. , & Curtis, N. (2020). Coronavirus infections in children including COVID‐19: An overview of the epidemiology, clinical features, diagnosis, treatment and prevention options in children. Pediatric Infectious Disease Journal, 39(5), 355. 10.1097/INF.0000000000002660 PMC7158880 32310621

