# Supplementary material for: Targeting SARS‐CoV‐2 with Chaga mushroom: An in silico study toward developing a natural antiviral compound
Source: Food Sci Nutr. 2021 Oct 20;9(12):6513–23. doi: 10.1002/fsn3.2576 (PMC8645752; doi:10.1002/fsn3.2576)
Supplement: Supplementary file 1 — Fig S1 [file FSN3-9-6513-s002.pdf]

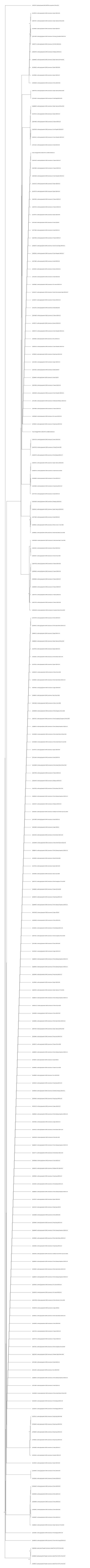
AID16716.1 spike glycoprotein Bat SARS-like coronavirus China 2012
QIU78872.1 surface glycoprotein SARS coronavirus-2 Spain 2020-03-06
93
QIM47457.1 surface glycoprotein SARS coronavirus-2 Spain Valencia 04-Mar-2020
95
QIU78848.1 surface glycoprotein SARS coronavirus-2 Spain 2020-02-27
QJF11935.1 surface glycoprotein SARS coronavirus-2 Germany Dusseldorf 2020-03-14
QIS60774.1 surface glycoprotein SARS coronavirus-2 USA WA 2020-03-16
QJD23270.1 surface glycoprotein SARS coronavirus-2 Malaysia 2020-03-14
QIQ08800.1 surface glycoprotein SARS coronavirus-2 Spain Valencia 26-Feb-2020
QIU78824.1 surface glycoprotein SARS coronavirus-2 Spain 2020-03-09
QIU78860.1 surface glycoprotein SARS coronavirus-2 Spain 2020-02-27
QJG65949.1 surface glycoprotein SARS coronavirus-2 China 2020-02-06
QIM47476.1 surface glycoprotein SARS coronavirus-2 Spain Valencia 08-Mar-2020
QJC19491.1 surface glycoprotein SARS coronavirus-2 India Rajkot2020-04-05
QIQ08820.1 surface glycoprotein SARS coronavirus-2 Spain Valencia 02-Mar-2020
QIU78719.1 surface glycoprotein SARS coronavirus-2 Spain 2020-03-10
QJD47800.1 surface glycoprotein SARS coronavirus-2 Taiwan 2020-03-14
QJD23153.1 surface glycoprotein SARS coronavirus-2 zech Republic 2020-03-19
QJD23141.1 surface glycoprotein SARS coronavirus-2 Czech Republic 2020-03-22
QJF11812.1 surface glycoprotein SARS coronavirus-2 India 2020-04-08
hCoV-19 Egypt NRC-03 2020 EPI ISL 430819 2020-03-18
QJD47812.1 surface glycoprotein SARS coronavirus-2 Taiwan 2020-03-17
QJD47884.1 surface glycoprotein SARS coronavirus-2 Taiwan 2020-03-18
QJD23189.1 surface glycoprotein SARS coronavirus-2 Czech Republic 2020-03-19
QJC21017.1 surface glycoprotein SARS coronavirus-2 Spain 2020-03-15
QIU78779.1 surface glycoprotein SARS coronavirus-2 Spain 2020-03-10
QJD47824.1 surface glycoprotein SARS coronavirus-2 Taiwan 2020-03-17
QJD47872.1 surface glycoprotein SARS coronavirus-2 Taiwan 2020-03-18
QIU78707.1 surface glycoprotein SARS coronavirus-2 Spain 2020-03-09
QIX12148.2 surface glycoprotein SARS coronavirus-2 France 2020-03
QJF77858.1 surface glycoprotein SARS coronavirus-2 India 2020-03-11
QJD47836.1 surface glycoprotein SARS coronavirus-2 Taiwan 2020-03-17
QIK50427.1 surface glycoprotein SARS coronavirus-2 USA CA San Diego 2020-03-11
QJD23201.1 surface glycoprotein SARS coronavirus-2 Czech Republic 2020-03-17
QJF77882.1 surface glycoprotein SARS coronavirus-2 India 2020-03-20
QIZ16535.1 surface glycoprotein SARS coronavirus-2 Greece 2020-03-18
QJF11824.1 surface glycoprotein SARS coronavirus-2 India 2020-04-06
QJD20861.1 surface glycoprotein SARS coronavirus-2 Sri Lanka 2020-03-19
QIZ15537.1 surface glycoprotein SARS coronavirus-2 South Africa KwaZulu-Natal 2020-03-07
QIZ16547.1 surface glycoprotein SARS coronavirus-2 Greece 2020-03-18
QJF11872.1 surface glycoprotein SARS coronavirus-2 India 2020-04-06
QJD47860.1 surface glycoprotein SARS coronavirus-2 Taiwan 2020-03-17
QIZ16571.1 surface glycoprotein SARS coronavirus-2 Greece 2020-03-18
QJD23177.1 surface glycoprotein SARS coronavirus-2 Czech Republic 2020-03-18
QIS60288.1 surface glycoprotein SARS coronavirus-2 Peru 2020-03-10
QJD23213.1 surface glycoprotein SARS coronavirus-2 Czech Republic 2020-03-18
QIT08292.1 surface glycoprotein SARS coronavirus-2 Hong Kong 2020-03-18
QJC21005.1 surface glycoprotein SARS coronavirus-2 Spain 2020-03-15
QJC21051.1 surface glycoprotein SARS coronavirus-2 Serbia 2020-04
QIT06999.1 surface glycoprotein SARS coronavirus-2 Israel 2020-03
QJD47848.1 surface glycoprotein SARS coronavirus-2 Taiwan 2020-03-16
QJD23165.1 surface glycoprotein SARS coronavirus-2 Czech Republic 2020-03-21
QJF11995.1 surface glycoprotein SARS coronavirus-2 Netherlands Milheeze 2020-04-24
QJD47896.1 surface glycoprotein SARS coronavirus-2 Taiwan 2020-03-18
QJD20849.1 surface glycoprotein SARS coronavirus-2 Sri Lanka 2020-03-10
QIT08304.1 surface glycoprotein SARS coronavirus-2 Hong Kong 2020-03-16
hCoV-19 Egypt NRC-01 2020 EPI ISL 430820 2020-03-18
QJD47718.1 surface glycoprotein SARS coronavirus-2 aiwan 2020-02-04
QHU79173.2 surface glycoprotein SARS coronavirus-2 Finland 29-Jan-2020
QIU81873.2 surface glycoprotein SARS coronavirus-2 China Beijing 2020-01-27
QIS30145.1 surface glycoprotein SARS coronavirus-2 Spain Valencia 2020-03-06
QHZ00379.1 surface glycoprotein SARS coronavirus-2 South Korea Jan-2020
QJG65956.1 surface glycoprotein SARS coronavirus-2 China 2020-02-12
QJC20993.1 surface glycoprotein SARS coronavirus-2 Hong Kong 2020-01-22
QJF77870.1 surface glycoprotein SARS coronavirus-2 India 2020-03-16
QJD23249.1 surface glycoprotein SARS coronavirus-2 Malaysia 2020-03-20
QIS30135.1 surface glycoprotein SARS coronavirus-2 Spain Valencia 2020-03-02
QJF77846.1 surface glycoprotein SARS coronavirus-2 India 2020-03-01
QIA20044.1 surface glycoprotein SARS coronavirus-2 China Yunnan 17-Jan-2020
QIA98583.1 surface glycoprotein SARS coronavirus-2 India Kerala State 31-Jan-2020
QHS34546.1 surface glycoprotein SARS coronavirus-2 India Kerala State 27-Jan-2020
QJA41641.1 surface glycoprotein SARS coronavirus-2 Brazil 2020-03-18
QIO04367.1 surface glycoprotein SARS coronavirus-2 China 20-Jan-2020
QJD47728.1 surface glycoprotein SARS coronavirus-2 Taiwan 2020-03-05
QJD20632.1 surface glycoprotein SARS coronavirus-2 Taiwan 2020-02-26
QJD20656.1 surface glycoprotein SARS coronavirus-2 Taiwan 2020-02-27
QJD20644.1 surface glycoprotein SARS coronavirus-2 Taiwan 2020-02-27
92
QJD47752.1 surface glycoprotein SARS coronavirus-2 Taiwan 2020-03-10
QJD47740.1 surface glycoprotein SARS coronavirus-2 Taiwan 2020-03-09
QHR84449.1 surface glycoprotein SARS coronavirus-2 Australia Victoria 25-Jan-2020
QJF74879.1 surface glycoprotein SARS coronavirus-2 China 2020-02-24
QIS30042.1 surface glycoprotein SARS coronavirus-2 China Hubei Wuhan 2020-01-25
QIB84673.1 surface glycoprotein SARS coronavirus-2 Nepal 2020-01-13
QIQ08830.1 surface glycoprotein SARS coronavirus-2 Spain Valencia 04-Mar-2020
QIU78743.1 surface glycoprotein SARS coronavirus-2 Spain 2020-03-10
QIU81801.1 surface glycoprotein SARS coronavirus-2 China Wuhan 2019-12-30
QIU78791.1 surface glycoprotein SARS coronavirus-2 Spain 2020-03-10
QHO62107.1 surface glycoprotein SARS coronavirus-2 China 02-Jan-2020
QIS29994.1 surface glycoprotein SARS coronavirus-2 China Hubei Wuhan 2020-01-26
BCB97901.1 surface glycoprotein SARS coronavirus-2 Japan 2020-03-09
QIA98554.1 surface glycoprotein SARS coronavirus-2 Italy 30-Jan-2020
QHN73810.1 surface glycoprotein SARS coronavirus-2 China 11-Jan-2020
QHZ00358.1 surface glycoprotein SARS coronavirus-2 China Hangzhou 20-Jan-2020
QIE07451.1 surface glycoprotein SARS coronavirus-2 china Guangdong Guangzhou 05-Feb-2020
QIQ68674.1 surface glycoprotein SARS coronavirus-2 China Zhejiang Hangzhou 2020-01-21
QHU36834.1 surface glycoprotein SARS coronavirus-2 China Hubei Wuhan 30-Dec-2019
QHU36864.1 surface glycoprotein SARS coronavirus-2 China Hubei Wuhan 01-Jan-2020
QIU78731.1 surface glycoprotein SARS coronavirus-2 Spain 2020-03-09
QJF11848.1 surface glycoprotein SARS coronavirus-2 India 2020-04-10
QHU36854.1 surface glycoprotein SARS coronavirus-2 China Hubei Wuhan 30-Dec-2019
QJD47788.1 surface glycoprotein SARS coronavirus-2 Taiwan 2020-03-14
QJD23225.1 surface glycoprotein SARS coronavirus-2 Malaysia 2020-03-18
QIU81789.1 surface glycoprotein SARS coronavirus-2 China Wuhan 2019-12-30
QIQ68564.1 surface glycoprotein SARS coronavirus-2 China Zhejiang Hangzhou 2020-01-23
QJD23237.1 surface glycoprotein SARS coronavirus-2 Malaysia 2020-03-20
QIK50448.1 surface glycoprotein SARS coronavirus-2 VietNam Ho Chi Minh city 22-Jan-2020
QJF11860.1 surface glycoprotein SARS coronavirus-2 India 2020-04-10
BCB15090.1 surface glycoprotein SARS coronavirus-2 Japan 2020-01
QIU81765.1 surface glycoprotein SARS coronavirus-2 China Wuhan 2019-12-30
QHU36844.1 surface glycoprotein SARS coronavirus-2 China Hubei Wuhan 30-Dec-201
QIQ68574.1 surface glycoprotein SARS coronavirus-2 China Zhejiang Hangzhou 2020-01-23
QIG55994.1 surface glycoprotein SARS coronavirus-2 Brazil 28-Feb-2020
QIU78755.1 surface glycoprotein SARS coronavirus-2 Spain 2020-03-09
QIC50498.1 surface glycoprotein SARS coronavirus-2 Italy 31-Jan-2020
QIE07471.1 surface glycoprotein SARS coronavirus-2 China Guangzhou 27-Jan-2020
QIA98606.1 surface glycoprotein SARS coronavirus-2 Taiwan 05-Feb-2020
QIG55875.1 surface glycoprotein SARS coronavirus-2 Hong Kong 2020-01-24
QIQ68694.1 surface glycoprotein SARS coronavirus-2 China Zhejiang Hangzhou 2020-01-21
BCD57643.1 surface glycoprotein SARS coronavirus-2 Japan 2020-03
QJG65948.1 surface glycoprotein SARS coronavirus-2 China 2020-02-03
QIU81861.1 surface glycoprotein SARS coronavirus-2 China Beijing 2020-01-28
QIE07461.1 surface glycoprotein SARS coronavirus-2 China Guangzhou 29-Jan-2020
QJF11896.1 surface glycoprotein SARS coronavirus-2 China 2020-03-06
BCA87361.1 surface glycoprotein SARS coronavirus-2 Japan 2020-02-10
QIQ68634.1 surface glycoprotein SARS coronavirus-2 China Zhejiang Hangzhou 2020-01-25
QIQ68684.1 surface glycoprotein SARS coronavirus-2 China Zhejiang Hangzhou 2020-01-21
QIG55895.1 surface glycoprotein SARS coronavirus-2 Hong Kong 2020-01-27
QIU78803.1 surface glycoprotein SARS coronavirus-2 Spain 2020-03-08
QIQ08790.1 surface glycoprotein SARS coronavirus-2 Spain Valencia 27-Feb-2020
QIQ68614.1 surface glycoprotein SARS coronavirus-2 China Zhejiang Hangzhou 2020-01-21
QHO62112.1 surface glycoprotein SARS coronavirus-2 China 02-Jan-2020
QJG65955.1 surface glycoprotein SARS coronavirus-2 China 2020-02-09
QIS30006.1 surface glycoprotein SARS coronavirus-2 China Hubei Wuhan 2020-01-26
QIM47467.2 surface glycoprotein SARS coronavirus-2 Spain Valencia 05-Mar-2020
QIG55885.1 surface glycoprotein SARS coronavirus-2 Hong Kong 2020-01-24
QIK50417.1 surface glycoprotein SARS coronavirus-2 Taiwan 25-Jan-2020
QIQ68604.1 surface glycoprotein SARS coronavirus-2 China Zhejiang Hangzhou 2020-01-21
QIT06987.1 surface glycoprotein SARS coronavirus-2 Israel 2020-02
QIA98596.1 surface glycoprotein SARS coronavirus-2 Taiwan 31-Jan-2020
QIU80900.1 surface glycoprotein SARS coronavirus-2 Iran 2020-03-09
QIG55915.1 surface glycoprotein SARS coronavirus-2 Hong Kong 2020-01-29
QIS30054.2 surface glycoprotein SARS coronavirus-2 Colombia Antioquia 2020-03-11
QIG55935.1 surface glycoprotein SARS coronavirus-2 Hong Kong 2020-01-29
BCA87371.1 surface glycoprotein SARS coronavirus-2 Japan 2020-02-10
QIQ68664.1 surface glycoprotein SARS coronavirus-2 China Zhejiang Hangzhou 2020-01-25
BCB97891.1 surface glycoprotein SARS coronavirus-2 Japan 2020-02-14
QIU81813.1 surface glycoprotein SARS coronavirus-2 China Wuhan 2019-12-30
QHD43416.1 surface glycoprotein SARS coronavirus-2 China Dec-2019
QIQ68624.1 surface glycoprotein SARS coronavirus-2 China Zhejiang Hangzhou 2020-01-25
QIU81777.1 surface glycoprotein SARS coronavirus-2 China Wuhan 2019-12-30
QHZ00369.1 surface glycoprotein SARS coronavirus-2 china 2020-01-22
QIS60276.1 surface glycoprotein SARS coronavirus-2 Pakistan KPK 2020-03-12
QIG55925.1 surface glycoprotein SARS coronavirus-2 Hong Kong 2020-01-27
QIU81825.1 surface glycoprotein SARS coronavirus-2 China Beijing 2020-01-24
QIQ68554.1 surface glycoprotein SARS coronavirus-2 China Zhejiang Hangzhou 2020-01-23
QIU78767.1 surface glycoprotein SARS coronavirus-2 Spain 2020-03-02
QIK02132.1 surface glycoprotein SARS coronavirus-2 Hong Kong 2020-01
QJG65950.1 surface glycoprotein SARS coronavirus-2 China 2020-02-06
QIG55865.1 surface glycoprotein SARS coronavirus-2 Hong Kong 2020-01-23
QIQ68584.1 surface glycoprotein SARS coronavirus-2 China Zhejiang Hangzhou 2020-01-24
QIS29982.1 surface glycoprotein SARS coronavirus-2 China Hubei Wuhan 2020-01-10
QIG55905.1 surface glycoprotein SARS coronavirus-2 Hong Kong 2020-01-27
QIK50438.1 surface glycoprotein SARS coronavirus-2 VietNam Ho Chi Minh city 22-Jan-2020
QIQ68594.1 surface glycoprotein SARS coronavirus-2 China Zhejiang Hangzhou 2020-01-25
QIS30030.1 surface glycoprotein SARS coronavirus-2 China Hubei Wuhan 2020-01-25
QIQ68644.1 surface glycoprotein SARS coronavirus-2 China Zhejiang Hangzhou 2020-01-22
QJD20837.1 surface glycoprotein SARS coronavirus-2 Sri Lanka 2020-03-16
QJD20873.1 surface glycoprotein SARS coronavirus-2 Sri Lanka 2020-03-31
QHN73795.1 surface glycoprotein SARS coronavirus-2 China Shenzhen 10-Jan-2020
BCD58753.1 surface glycoprotein SARS coronavirus-2 Japan 2020-02
QIS30018.1 surface glycoprotein SARS coronavirus-2 China Hubei Wuhan 2020-01-26
QJG65953.1 surface glycoprotein SARS coronavirus-2 China 2020-02-08
QJD47764.1 surface glycoprotein SARS coronavirus-2 Taiwan 2020-03-13
QJD47776.1 surface glycoprotein SARS coronavirus-2 Taiwan 2020-03-13
QIE07481.1 surface glycoprotein SARS coronavirus-2 China Guangzhou 29-Jan-2020
QIQ22760.1 surface glycoprotein SARS coronavirus-2 Pakistan Gilgit 04-Mar-2020
QJF11836.1 surface glycoprotein SARS coronavirus-2 India 2020-04-14
QIX12195.1 surface glycoprotein SARS coronavirus-2 Iran 2020-03-09
QIQ68654.1 surface glycoprotein SARS coronavirus-2 China Zhejiang Hangzhou 2020-01-22
QJF11884.1 surface glycoprotein SARS coronavirus-2 India 2020-04-14
QHU36824.1 surface glycoprotein SARS coronavirus-2 China Hubei Wuhan 23-Dec-2019
QIU81849.1 surface glycoprotein SARS coronavirus-2 China Beijing 2020-01-28
QIU81837.1 surface glycoprotein SARS coronavirus-2 China Beijing 2020-01-25
QIT07011.1 surface glycoprotein SARS coronavirus-2 Hong Kong 2020-03-08
QIT08280.1 surface glycoprotein SARS coronavirus-2 Hong Kong 2020-02-25
90
QIT08268.1 surface glycoprotein SARS coronavirus-2 Hong Kong 2020-02-24
QIT08256.1 surface glycoprotein SARS coronavirus-2 Hong Kong 2020-02-26
QIZ16509.1 surface glycoprotein SARS coronavirus-2 Turkey 2020-03-17
QIC53204.1 surface glycoprotein SARS coronavirus-2 Sweden 2020-02-07
QIU78825.1 surface glycoprotein SARS coronavirus-2 Spain 2020-03-09
QJG65952.1 surface glycoprotein SARS coronavirus-2 China 2020-02-06
QIZ16559.1 surface glycoprotein SARS coronavirus-2 Greece 2020-03-18
QJG65947.1 surface glycoprotein SARS coronavirus-2 China 2020-02-03
QJG65954.1 surface glycoprotein SARS coronavirus-2 China 2020-01-23
QJG65958.1 surface glycoprotein SARS coronavirus-2 China 2020-02-18
100
QJG65951.1 surface glycoprotein SARS coronavirus-2 China 2020-02-06
QJG65957.1 surface glycoprotein SARS coronavirus-2 China 2020-02-15
QIQ08810.1 surface glycoprotein SARS coronavirus-2 Spain Valencia 27-Feb-2020
QIU81885.1 surface glycoprotein SARS coronavirus-2 China Beijing 2020-01-29
QIU80913.1 surface glycoprotein SARS coronavirus-2 China Anhui Fuyang 2020-03-10
QIQ54048.1 spike protein Pangolin coronavirus isolate PCoV GX-P2V China 2018
89
QIA48632.1 spike glycoprotein Pangolin coronavirus isolate PCoV GX-P5L China 2017
100
QIA48614.1 spike glycoprotein Pangolin coronavirus isolate PCoV GX-P4L China 2017
